# Development of bio-based fine chemical production through synthetic bioengineering

**DOI:** 10.1186/s12934-014-0173-5

**Published:** 2014-12-14

**Authors:** Kiyotaka Y Hara, Michihiro Araki, Naoko Okai, Satoshi Wakai, Tomohisa Hasunuma, Akihiko Kondo

**Affiliations:** 1 Organization of Advanced Science and Technology, Kobe University, Nada, Kobe, Japan; 2 Department of Chemical Science and Engineering, Graduate School of Engineering, Kobe University, 1-1 Rokkodaicho, Nada, Kobe, 657-8501 Japan

**Keywords:** Fine chemical, Synthetic bioengineering, Metabolic engineering, Enzymatic synthesis, Microbial fermentation, Bioinformatics

## Abstract

Fine chemicals that are physiologically active, such as pharmaceuticals, cosmetics, nutritional supplements, flavoring agents as well as additives for foods, feed, and fertilizer are produced by enzymatically or through microbial fermentation. The identification of enzymes that catalyze the target reaction makes possible the enzymatic synthesis of the desired fine chemical. The genes encoding these enzymes are then introduced into suitable microbial hosts that are cultured with inexpensive, naturally abundant carbon sources, and other nutrients. Metabolic engineering create efficient microbial cell factories for producing chemicals at higher yields. Molecular genetic techniques are then used to optimize metabolic pathways of genetically and metabolically well-characterized hosts. Synthetic bioengineering represents a novel approach to employ a combination of computer simulation and metabolic analysis to design artificial metabolic pathways suitable for mass production of target chemicals in host strains. In the present review, we summarize recent studies on bio-based fine chemical production and assess the potential of synthetic bioengineering for further improving their productivity.

## Introduction

Physiologically active fine chemicals such as pharmaceuticals, cosmetics, nutritional supplements, flavoring agents as well as additives for foods, feed, and fertilizer are produced enzymatically or through microbial fermentation. Although many of these compounds are present naturally, few are commercially available, because most are present in low abundance and may be difficult and expensive to purify. These disadvantages are overcome by bio-based fine chemical synthesis.

**Figure 1:**
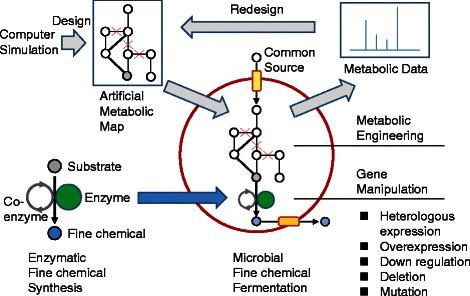
**Bio-based fine chemical production through synthetic bioengineering.** Enzymes convert substrates to the fine chemical of interest with or without a coenzyme. The enzymatic synthesis system is introduced into a microbial host strain to develop a microbial cell factory (blue arrow). The microbial system converts a common source into various fine chemicals, and they are accumulated in cells or in the medium. The productivity of a microbial cell factory is improved by genetic engineering of metabolic pathways (e.g. heterologous expression, overexpression, down-regulation, deletion, or mutation) according to an artificial metabolic map designed by computer simulation. Further, synthetic bioengineering (gray arrows) improves productivity by additional metabolic engineering according to the artificial map redesigned by the metabolic data of the microbial cell factory.

Bio-based fine chemical production is summarized in Figure 1. The enzymes are isolated from diverse organisms and are used in purified form *in vitro* or expressed by a suitable host cell.

The advantage of microbial fermentation is that the supply of components required for growth of the host strain and synthesis of the product can be derived from inexpensive sources of carbon, nitrogen, trace elements, and energy [1]. In particular, coproduction of several fine chemicals from common carbon sources is more economical. The conditional requirements to fulfill the price advantages for the production of target fine chemicals are rapid cell growth to high density, and high cellular content and easy extraction of target fine chemicals. The bio-production of fine chemicals is typically performed at lower temperatures compared with those required for chemical syntheses, and important advantages of bio-based fine chemical production are cost-effectiveness and the use of processes that are not hazardous to the environment.

Synthetic bioengineering represents a recently developed novel approach to create optimized microbial cell factories for efficient production of target compounds through fermentation (Figure 1). Synthetic bioengineering is achieved using genetic engineering strategies designed according to artificial metabolic maps generated by computer simulation. Metabolomic data are critical to redesign a rational artificial metabolic map in which metabolic sources flow efficiently into the target compound. The concentrations of enzyme and substrates are readily controlled in an enzymatic reaction mixture; however, this is difficult in fermentations because precursors may be shunted through different metabolic pathways. Thus, synthetic bioengineering plays a critical role in controlling metabolic pathways to supply the optimal substrate ratios. To develop highly productive microbial fermentation systems for producing fine chemicals, the genes encoding the required enzymes are introduced into an appropriate host strain (Figure 1). Thus, the key for selecting the host strain, target metabolic pathways, or both to improve the production of fine chemicals by fermentation is the ability to genetically engineer modifications to the relevant metabolic pathways. *Escherichia coli* is often selected as the first candidate for producing target enzymes because of its well-developed genetic engineering system and its ability to express high levels of genes encoding target enzymes. In contrast, microorganisms such as *Saccharomyces cerevisiae*, *Bacillus* strains, *Streptomyces* strains, *Corynebacterium glutamicum,* and *Aspergillus oryzae* are selected as a host for fermentations depending on their specific metabolic pathways that are required to synthesize target products.

Here we summarize recently developed, well-characterized bio-based systems for producing the compounds as follows: γ-aminobutyric acid (GABA), isoprenoids, aromatics, peptides, polyphenols, and oligosaccharides (Table 1). The developmental stages of these systems are different, and they illustrate the great potential of synthetic bioengineering approaches for producing all bio-based fine chemicals in the future.

**Table 1:** **Bio-based fine chemicals**

**Chemical category**	**Example structure**	**Function**	**Production types**
γ-aminobutyric acid (GABA)	GABA	Cosmetics	Microbial fermentation
		Nutritional supplement	
		Food additive	
Isoprenoid	Isoprene	Medicine	Microbial fermentation
		Cosmetics	
		Nutritional supplement	
		Flavoring agent	
		Food additive	
		Feed additive	
		Fertilizer additive	
Aromatic compound	Cinnamic acid	Medicine	Microbial fermentation
		Cosmetics	
		Nutritional supplement	
		Flavoring agent	
		Food additive	
Alkaloid	Reticuline	Medicine	Microbial fermentation
Peptide	Glutathione	Medicine	Enzymatic production/Microbial fermentation
		Cosmetics	
		Nutritional supplement	
		Food additive	
		Feed additive	
		Fertilizer additive	
Polyphenol	Resveratrol	Medicine	Microbial fermentation
		Cosmetics	
		Nutritional supplement	
		Flavoring agent	
		Food additive	
		Feed additive	
		Fertilizer additive	
Oligosaccharide	2’-fucosyllactose	Medicine	Enzymatic production
		Cosmetics	
		Nutritional supplement	
		Flavoring agent	
		Food additive	
		Feed additive	
		Fertilizer additive	

### GABA

The microbiological production of GABA serves as an excellent first example of how a system can be improved to increase yields (Table 2). GABA is an amino acid, which is not present naturally in proteins, that is synthesized by microorganisms, animals, and plants [2]. GABA functions as a neurotransmitter signals decreases blood pressure [3] and is used in functional foods and pharmaceuticals [4]. GABA, which was originally identified in traditional fermented foods such as cheese, yogurt [5] and kimchi [6], is synthesized through the alpha-decarboxylation of L-glutamate catalyzed by glutamate decarboxylase (GAD, EC 4.1.1.15) [2]. GABA is produced by lactic acid bacteria (LABs) such as *Lactobacillus paracasei* [7], *L. buchneri* [6], and *L. brevis* [8,9] (Table 2), and the latter produces high yields of GABA through fed-batch processes [10].

**Table 2:** **Microbial fermentation of GABA**

**Strains**	**Source or engineered phenotype**	**Substrates**	**Yield (g/L)**	**Scale (L)**	**Reference**
*L. paracasei* NFRI 7415	Isolated from fermented crucians	MSG	31.1	-	Komatsuzaki et al., 2005 [7]
*L. buchneri* MS	Isolated from Kimchi	MSG, Saccharides	25.8	-	Cho et al., 2007 [6]
*S. salivarius* subsp. *thermophilus* Y2	Starter for yoghurt and cheese	MSG	7.98	0.4	Yang et al., 2008 [5]
*L. brevis* NCL912	Isolated from Paocai	MSG	35.6	0.1	Li et al., 2010 [4]
		L-glutamate (fed-batch fermentation)	102.8	3.0	Li et al., 2010 [10]
TCCC13007	Isolated from pickles	MSG (2-step fermentation)	61	3.0	Zhang et al., 2012 [8]
*E. coli*	*gadB* (*L. lactis*)	MSG	76.2	1.5	Park et al., 2013 [11]
*C. glutamicum*	*gadRCB2* (*L. brevis*)	Glucose	2.15	0.02	Shi et al., 2011 [12]
	*gadB* (*E. coli*)	Glucose	12.3	0.02	Takahashi et al., 2012 [13]
	*gadB1B2* (*L. brevis*)	Glucose, urea	27.1	1.2	Shi et al., 2013 [14]
	Δ*pknG*, *gadB* (*E. coli*)	Glucose	31.1	0.02	Okai et al., 2014 [15]

*Corynebacterium glutamicum* is an important industrial microorganism because of its ability to produce high levels of L-glutamate, and recombinant strains of *C. glutamicum* that express GADs from *L. brevis* [12,14] or *Escherichia coli* [13] produce GABA from glucose (Table 2). Disrupting the gene that encodes protein kinase G affects the function of 2-oxoglutarate dehydrogenase in the TCA cycle, alters metabolic flux toward glutamate [16], and enhances the yields of GABA produced by a GAD-expressing strain of *C. glutamicum* [15]. Because *C. glutamicum* is generally recognized as safe, the system for GABA fermentation can be applied to the production of GABA as a component of food additives and pharmaceuticals.

### Isoprenoids

Isoprenoids represent the most diverse group of natural products comprising more than 40,000 structurally distinct compounds that are present in all classes of living organisms. These molecules play key roles in respiration and electron transport, maintenance of membrane fluidity, hormone signaling, photosynthesis, antioxidation as well as subcellular localization and regulation of protein activities [17]. Certain isoprenoids such as carotenoids are produced commercially as nutritional and medicinal additives [18].

**Figure 2:**
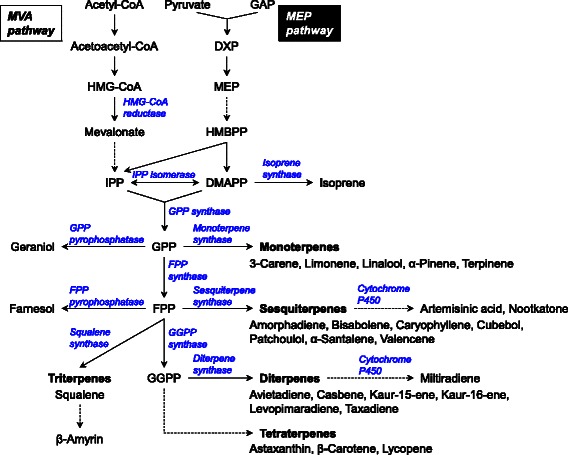
**Biosynthetic pathway of isoprenoids produced by recombinant microorganisms.** Abbreviations: DXP, 1-deoxy-D-xylulose-5-phosphate; MEP, 2-C-methyl-D-erythritol-4-phosphate; HMBPP, hydroxymethylbutenyl-4-diphosphate; IPP, isopentenyl diphosphate; DMAPP, dimethylallyl diphosphate; GPP, geranyl diphosphate; FPP, farnesyl diphosphate; GGPP, geranylgeranyl diphosphate; HMG-CoA, 3-hydroxy-3-methylglutaryl-CoA.

Despite their enormous structural diversity, isoprenoids are biologically synthesized through consecutive condensations of five-carbon precursors, isopentenyl diphosphate (IPP), and its allyl isomer dimethylallyl diphosphate (DMAPP) (Figure 2). IPP and DMAPP are synthesized via either the mevalonate (MVA) pathway in most eukaryotes or the 2-*C*-methyl-D-erythrito-l,4-phosphate (MEP) pathway in prokaryotes. In higher plants, the MVA and MEP pathways function in the cytosol and plastid, respectively [19-21]. The five-carbon precursors are condensed by prenyltransferase to form prenyl pyrophosphates such as geranyl pyrophosphate (GPP), farnesyl pyrophosphate (FPP), geranylgeranyl pyrophosphate (GGPP), and several polyprenyl pyrophosphates [17]. The prenyl pyrophosphates are converted into monoterpenes, sesquiterpenes, diterpenes, triterpenes, tetraterpenes, and polyprenyl side chains. The chemical diversity of isoprenoids is determined by specific terpene synthases and terpene-modifying enzymes, particularly cytochromes P450 [22].

Various synthetic bioengineering approaches improve isoprenoid production by microorganisms such as *E. coli* and *S. cerevisiae*. Examples include the synthesis of triterpenoids amorpha-4,11-diene and artemisinic acid, precursors of the antimalarial agent artemisinin and the precursor of the major antineoplastic agent taxol diterpenoid taxa-4(5),11(12)-diene [23-31]. The tetraterpenoids (carotenoids) such as astaxanthin are also synthesized by synthetic bioengineering approaches [32,33].

Isoprene, the simplest isoprenoid, is used to synthesize pharmaceuticals, pesticides, fragrances, and synthetic rubber. *E. coli* strains engineered to express the *Poplus alba* gene (*IspS*) encoding isoprene synthase and the *S. cerevisiae* MVA pathway genes produce 532 mg/L of isoprene under fed-batch conditions [34]. A strain of the cyanobacterium *Synechocystis*, which was engineered to express *IspS* from kudzu, synthesizes isoprene photosynthetically [35].

Monoterpenes are used as aromatic additives in food, wine, and cosmetics. Certain monoterpenes exhibit antimicrobial, antiparasitic, and antiviral activities [36]. In *S. cerevisiae*, geraniol and linalool are produced from GPP by the expression of genes encoding geraniol synthase and linalool synthase (LIS), respectively [37-40]. Expression of the gene encoding *Picea abies* 3-carene cyclase in *E. coli* generates a range of monoterpenes, including α-pinene, myrcene, sabinene, 3-carene, γ-terpinene, limonene, β-phellandrene, α-terpinene, and terpinolene [41].

Sesquiterpenes exhibit anticancer, cytotoxic, and antibiotic properties as well as their characteristic flavors and aromas, making them industrially relevant compounds [17]. Valencene, cubebol, patchoulol, and α-santalene are produced by expressing heterologous sesquiterpene synthase genes in *S. cerevisiae* [42-47]. Coexpression of the genes encoding valencene synthase gene and a P450 mono-oxygenase in *S. cerevisiae* generates nootkatone that is industrially produced as a flavoring agent and fragrance [48]. The diterpenoid levopimaradiene is produced at high yield (700 mg/L) through combinatorial protein engineering of plant-derived GGPP synthase and levopimaradiene synthase expressed by an *E. coli* strain with enhanced carbon flux toward IPP and DMAPP [49]. Miltiradiene, the precursor of tanshinones that belongs to a group of bioactive diterpenoids present in the Chinese medicinal herb *Salvia miltiorrhizha*, accumulates in a *S. cerevisiae* strain that expresses genes encoding *S. miltiorrhiza* copalyl diphosphate synthase and kaurene synthases homolog [50,51]. Few attempts were made to metabolically engineer triterpene production, because the genes encoding components of its biosynthetic pathway are unknown; Kirby et al. isolated a gene encoding β-amyrin synthase from *Artemisia annua* and used it to produce β-amyrin in *S. cerevisiae* [52].

Elevating the levels of precursor pools based on improving carbon flux are efficient strategies to enhance isoprenoid production by recombinant microbial strains (Table 3). Scalcinati et al. adopted multiple metabolic engineering strategies for α-santalene production that were designed to increase precursor and cofactor supply by improving metabolic flux toward FPP and modifying the ammonium assimilation pathway, respectively [45,46]. The gene encoding 3-hydroxy-3-methyl-glutaryl-CoA reductase lacking its transmembrane region was expressed to avoid feedback regulation by sterols [45,46]. Repression of *ERG9* that encodes squalene synthase and the deletion of *LPP1* and *DPP1* that encode enzymes that dephosphorylate FPP minimized the formation of by-products such as sterols and farnesol. Efficient provision of acetyl-CoA, the precursor of the MVA pathway, was critical to improve α-santalene synthesis [47].

**Table 3:** **Strategies of synthetic bioengineering for the microbial production of isoprenoids**

**Compounds**	**Host strains**	**Genetic engineering** ^**a**^	**Strategy for flux control**	**Titer**	**Reference**
Isoprene	*E. coli*	**He:** *B. subtilis dxs*, *dxr* and *P. alba IspS*	-Improvement of MEP pathway flux	314 mg/L	Zhao et al., 2011 [30]
	*E. coli*	**He:** *P. alba IspS*	-Integration of heterologous MVA pathway	532 mg/L	Yang et al., 2012 [34]
		**Oe:** MVA pathway genes			
***Monoterpene***					
Carene	*E. coli*	**He:** *H. pluvialis* IPI isomerase and *P. abies* 3- carene cyclase genes	-Improvement of flux toward GPP	3 μg/L/OD_600_	Reiling et al., 2004 [41]
		**Oe:** *dxs*, *IspA* variant			
Geraniol	*S. cerevisiae*	**He:** *O. basiilcum* geraniol synthase gene	-Repression of FPP synthesis	5 mg/L	Fischer et al., 2011 [40]
		**Mu:** *ERG20*			
Linalool	*S. cerevisiae*	**He:** *C. breweri LIS*	-Improvement of MVA pathway flux	132.66 μg/L	Rico et al., 2010 [39]
		**Oe:** *tHMG1*			
***Sesquiterpene***					
Artemisinic acid (Amorpha-4,11-diene)	*E. coli*	**He:** *S. cerevisiae HMGS*, *tHMG1*, *ERG12*, *ERG8*, *MVD1*, *H. pluvialis ispA* and *A. annua ADS*	-Integration of heterologous MVA pathway	111.2 mg/L	Martin et al., 2003 [53]
			-Overexpression of FPP synthase gene		
		**Oe:** *atoB* and *idi*			
	*S. cerevisiae*	**He:** *A. annua ADS* and *CYP71AV1*	-Overexpression of tHMGR and FPP synthase genes	153 mg/L	Ro et al., 2006 [54]
		**Oe:** *tHMG1*, *ERG20 and Upc2-1*			
			-Up regulation of global transcription activity		
		**Dr:** *ERG9*			
			-Repression of squalene synthesis		
		**He:** *A. annua ADS*, *CYP71AV1*, *CPR1*, *CYB5*, *ALDH1* and *ADH1*	-Integration of heterologous MVA pathway	25 g/L	Paddon et al., 2013 [55]
			-Overexpression of tHMGR and FPP synthase genes		
			-Repression of		
			squalene synthesis		
		**Oe:** MVA pathway genes, *tHMGR*, and *ERG20*			
		**Dr:** *ERG9*			
Levopi-maradiene	*E. coli*	**He:** *G. biloba GGPPS* and *LPS*	-Improvement of flux toward IPP/DMAPP	700 mg/L	Leonard et al., 2010 [49]
		**Oe:** *dxs*, *idi*, *ispD* and *ispF*	-Combinatorial mutation in GGPP synthase and LPS		
Patchoulol	*S. cerevisiae*	**Oe:** *S. cerevisiae FPPS*/*P. cablin PTS* (chimera)	-Avoidance of intermediate loss	40.9 mg/L	Albertsen et al., 2011 [43]
			-Repression of squalene synthesis		
		**Dr:** *ERG9*			
α-Santalene	*S. cerevisiae*	**He:** *C. lansium* santalene synthase	-Overexpression of tHMGR and FPP synthase	92 mg/L	Scalcinati et al., 2012 [42]
		**Oe:** *tHMG1*, *FPPS*, *GDH2* and *Upc2-1*			
			-Increment of cofactor supply		
		**Dr:** *ERG9*			
		**De:** *GDH1*, *LPP1*, *DPP1*	-Up regulation of global transcription activity		
			-Repression of squalene synthesis		
			-Minimization of flux toward farnosol		
		**He:** *C. lansium* santalene synthase	-Improvement of flux toward acetoacetyl-CoA from ethanol	8.3 mg/L	Chen et al., 2013 [44]
			-Avoidance of acetyl-CoA consumption		
		**Oe:** *Adh2*, *Ald6*, *ACS* variant, *Erg10* and *tHMG1*			
		**De:** *CIT2* and *MLS1*			
Valencene	*S. cerevisiae*	**He:** *A. thaliana* FPP synthase and *C. sinensis* TPS1 genes in mitochondria	-Overexpression of tHMGR	1.5 mg/L	Farhi et al., 2011 [44]
			-Mitochondrial expression of FPP synthase and valencene synthase genes		
		**Oe:** *tHMG1*			
***Diterpene***					
Casbene	*E. coli*	**Oe:** *dxs*, *IspA* variant	-Improvement of flux toward GGPP	0.3 mg/L	Reiling et al., 2004 [41]
		**He:** *H. pluvialis* IPI isomerase and *R. communis* casbene cyclase genes			
Miltiradiene	*S. cerevisiae*	**He:** *S. acidocaldarium GGPPS* and *S. miltiorrhizha CPS* and *KSL*	-Overexpression of tHMGR, FPP synthase and GGPP synthase genes	8.8 mg/L	Dai et al., 2012 [47]
		**Oe:** *tHMG1*, *ERG20*, *BTS1* and *Upc2-1*	-Up regulation of global transcription activity		
Taxadiene	*E. coli*	**He:** *S. acidocaldariums* GGPP synthase and *T. chinensis* taxadiene synthase genes	-Improvement of MEP pathway flux	1 g/L	Ajikumar et al., 2010 [24]
			-Overexpression of GGPP synthase		
		**Oe:** *dxs*, *ispC*, *ispF*, *idi*, *tHMG1* and *Upc2-1*			
	*S. cerevisiae*	**He:** *S. acidocaldariums* GGPP synthase and *T. chinensis* taxadiene synthase genes	-Overexpression of tHMGR	8.7 mg/L	Engels et al., 2008 [31]
			-Up regulation of global transcription activity		
		**Oe:** *tHMG1* and *Upc2-1*			
***Triterpene***					
β-Amyrin	*S. cerevisiae*	**Oe:** *tHMG1*	-Overexpression of tHMGR	6 mg/L	Kirby et al., 2008 [52]
		**He:** *A. annua* β-amyrin synthase gene			
			-Repression of lanosterol synthesis		
		**Dr:** *ERG7*			

^a^Types of genetic engineering: He, Heterologous expression; Oe, Overexpression of self-cloning gene(s); Dr, down regulation; De, Deletion; Mu, Mutation.

### Aromatics

Aromatic compounds such as vanillin, cinnamic acid, *p*-hydroxycinnamic acid, and caffeic acid are used as flavoring agents or food ingredients (Table 4). Vanillin, which was originally extracted from cured seed pods of the orchid *Vanilla planifolia*, is mainly synthesized from petroleum oil or lignin. Alternatively, vanillin is produced by bioconversion of fossil carbon, guaiacol, eugenol, or isoeugenol [56]. Vanillin was produced from glucose by fermentation using an engineered strain of barker’s yeast [57]. To decrease the cytotoxic effects of converting intercellular 3-dehydroshikiminate to vanillin, genes encoding UDP-glucose transferase and *o*-methyltransferase were introduced into baker’s yeast to produce vanillyl glucoside (VG) [58]. Further, the Minimization of Metabolic Adjustments (MOMA) [59] and OptGene [60] algorithms were used to improve VG production in yeast strains [58,61].

**Table 4:** **Microbial fermentation of aromatic compounds**

**Compounds**	**Strains**	**Genetic engineering** ^**a**^	**Substrates**	**Yield (g/L)**	**Reference**
Vanillin	*S. pombe*	**He:** 3-dehydroshikiminate dehydratase (3DSD), Aryl carboxylic acid reductase (ACAR*, Nocardia sp.*), *o*-methyltransferase (OMT), UDP-glycosyltransferase (UGT, *A. thaliana*)	Glucose	0.065	Hansen et al., 2009 [51]
	*S. cervisiae*	**He:** 3DSD, ACAR, OMT, UGT			
Vanillin β-D-glucoside	*S. cervisiae*	**He:** 3DSD, ACAR, phosphopantetheine transferase (PPTase), hsOMT (*Homo sapiens*), UGT	Glucose	0.5	Brochado et al., 2010 [52]
		**De:** *pdc1gdh1*↑*GDH2*			
	*S. cervisiae*	**He:** ACAR, hsOMT,	Glucose	0.38	Brochado et al., 2013 [58]
		**Oe:** *GDH2*			
		**De:** *pdc1 gdh1 yprC*			
Cinnamic acid	*P. putida*	**He:** Phenylalanine ammmonia lyase (PAL, *Rhodosporidium toruloides*)	Glucose	0.74	Nijkamp et al., 2005 [62]
			Glycerol	0.8	
	*S. lividans*	**He:** PAL (*Streptomyces maritimus*)	Glucose	0.12	Noda et al., 2011 [60]
			Starch	0.46	
			Xylose	0.3	
			Xylan	0.13	
*p*-hydroxycinnamic acid (*p*-coumaric acid)	*S. cervisiae*	**He:** PAL/TAL (*Rhodotorula glutinis*), plant Cytochrome P450 (Cyt P450), Cyt reductase	Glucose		Vannelli et al., 2007 [62]
	*E. coli*	**He:** PAL/TAL (*R. glutinis*)		0.10	
	*S. lividans*	**He:** Tyrosine ammmonia lyase (TAL, *Rhodobacter sphaeroides*)	Glucose	0.75	Kawai et al., 2013 [63]
			Cellobiose	0.74	
		**He:** TAL (*R. sphaeroides*), Endoglucanase (*Thermobifida fusca*)	PASC	0.5	
	*P. putida*	**He:** PAL (*Rhodosporidium toruloides*)	Glucose	1.7	Nijkamp et al., 2007 [64]
		**Mu:** phenylalanine bradytrophic			
		**De:** *fcs*			
	*E. coli*	**He:** TAL (*Saccharothrix espanaensis*)	Glucose	0.97	Kang et al., 2012 [65]
		**Mu:** t*yrA* ^*fbr*^ *aroG* ^*fbr*^			
		**De:** *tyrR*			
Caffeic acid	*E. coli*	**He:** Cyt P450 (*Rhodopseudomonas palustris*)	*p*-hydroxycinnamic acid	2.8	Furuya et al., 2012 [66]
		**He, Mu:** Cyt P450	Cinnamic acid		
		**He:** TAL, 4-coumarate hydroxylase (Sam5, *S. espanaensis*),	Glucose	0.15	Kang et al., 2012 [65]
		**Mu:** *tyrA* ^fbr^ *aroG* ^fbr^			
		**De:** *tyrR*			
		**He:** TAL (*R. glutinis* codon-optimized), 4-coumarate: CoA ligase (4CL), 4-coumarate 3-hydroxylase (Coum3H, *S. espanaensis*)	Glucose	0.10	Zhang et al., 2013 [67]
			Xylose	0.07	
		**Mu:** *tyrA* ^fbr^ *aroG* ^fbr^			
		**De:** *tyrR pheA*			

^a^Types of genetic engineering: He, Heterologous expression; Oe, Overexpression of self-cloning gene(s); Mu, Mutation; De, Deletion.

Cinnamic acid is used as a cinnamon flavoring agent and is antibacterial. Although cinnamic acid occurs abundantly in plants as a precursor of phenylpropanoids, it is produced industrially using synthetic organic chemistry. Cinnamic acid is produced from sugar by phenylalanine-ammonia lyase (PAL, EC 4.3.1.24) expressed in a solvent-tolerant *Pseudomonas putida* strain [62] or by *Streptomyces lividans*, which is an ideal host, because its endogenous polyketide synthesis (PKS) pathways synthesize phenylpropanoids [64]. A phenylpropanoid, *p*-hydroxycinnamic acid (*p*-coumaric acid), a constituent of the plant cell wall, which is covalently linked to polysaccharides and lignins, acts as an antioxidant in humans [63]. *E. coli* and *S. cerevisiae* strains engineered to express PAL/tyrosine-ammonia lyase (TAL, EC 4.3.1.23) [68] or *P. putida* engineered to express PAL [69] produce *p*-hydroxycinnamic acid from glucose. Further, *p*-hydroxycinnamic acid can be produced from cellulose by *S. lividans* coexpressing TAL and endoglucanase (EG, EC 3.2.1. 4) [70].

### Alkaloids

**Figure 3:**
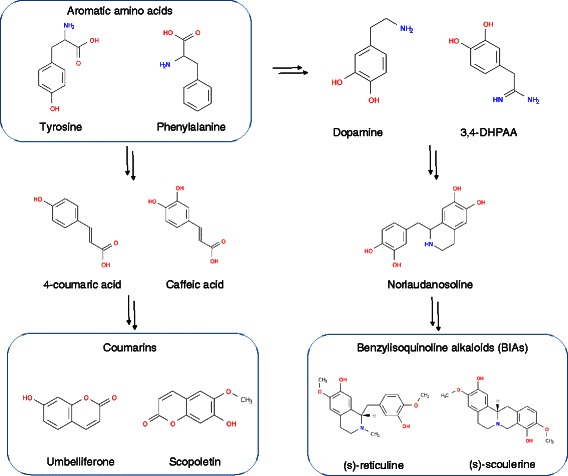
**Production of benzylisoquinoline alkaloids (BIAs) and coumarins from aromatic amino acids.** BIAs and coumarins are synthesized from aromatic amino acids (tyrosine and phenylalanine) through corresponding intermediates.

Alkaloids are nitrogen-containing compounds derived from amino acids such as histidine, lysine, ornithine, tryptophan, and tyrosine [71] that are present in plants. Most are used in biological and medicinal applications. They are mainly extracted from plants for practical use, but the yields are very low because low levels of alkaloids are produced by plants. Further, alkaloids consist of complex chemical backbones and structures with one or more chiral centers, which make it difficult to supply sufficient amounts of alkaloids for practical use through chemical synthesis. Therefore, development of alternative approaches is expected to characterize and engineer the biosynthetic pathways in microbial and plant cells. Benzylisoquinoline alkaloids (BIAs) such as (s)-reticuline and (s)-scoulerine, which are categorized into one of the major alkaloid subclasses, include the analgesics codeine and morphine, the antimicrobial berberine, and the anticancer drug noscapine (Figure 3 right). Both (s)-reticuline and (s)-scoulerine are synthesized through the production of (*R*, *S*)-norlaudanosoline from aromatic amino acids (tyrosine and phenylalanine). The increasing volume of information on the genome sequences of alkaloid-producing plants makes it possible to identify and engineer genes in biosynthetic pathways to produce BIAs in *E. coli* [72,73] and *S. cerevisiae* [74] through the production of (*R*, *S*)-norlaudanosoline from aromatic amino acids.

Minami et al. reconstructed the (*S*)-reticuline biosynthetic pathways in *E. coli* using monoamine oxidase (MAO) from *Micrococcus luteus* and four genes from *Coptis japonica* to produce (*S*)-reticuline from dopamine. They introduced the genes encoding BBE (berberine bridge enzyme) and CYP80G2 (plant cytochrome P450 enzyme) of *C. japonica* into *S. cerevisiae*, because active forms of certain plant enzymes cannot be expressed in *E. coli. S. cerevisiae* provides the advantage of compartmentalizing these proteins in the cytosol and endoplasmic reticulum (ER). The engineered *S. cerevisiae* is co-cultured with *E. coli* to produce BIA derivatives (*S*)-scoulerine and magnoflorine. Nakagawa et al. engineered the shikimate (SK) pathway in *E. coli* to increase the amount of L-tyrosine and produced (*S*)-reticuline from glucose or glycerol [73]. The BIA pathways downstream of the precursor (*R*, *S*)-norlaudanosoline were assembled in *S. cerevisiae* [74]. The expression levels of norcoclaurine 6-O-methyltransferase (6-OMT), coclaurine-N-methyltransferase (CNMT), and 3′-hydroxy- N-methylcoclaurine 4′-O-methyltransferase (4′-OMT), and a hydroxylation reaction catalyzed by cytochrome P450 80B1 (CYP80B1) derived from *Thalictrum flavum* or *Papaver somniferum* were optimized to produce (*S*)-reticuline. A human cytochrome P450 (CYP2D6) was expressed as well to produce the morphinan intermediate salutaridine [71]. Most recently, 10 genes from plant BIA pathways were introduced into *S. cerevisiae* to produce dihydrosanguinarine and its oxidized derivative sanguinarine from (*R,S*)-norlaudanosoline [75].

Coumarins are present in plants and are used as antibacterials, anticancer drugs, and anticoagulants. The biosynthetic pathways of coumarins diverge from that of phenylalanine/tyrosine as well as BIA (Figure 3 left). Recent findings that coumarin is formed by the action of two hydroxylases allowed reconstruction of its biosynthetic pathway in microbial cells. Lin et al. designed artificial biosynthetic pathways in *E. coli* [76] and produced scopoletin and umbelliferone from the corresponding phenylpropanoid acids, ferulic acid, caffeic acid, and 4-coumaric acid. They used TAL to produce scopoletin and umbelliferone from aromatic amino acids. Further work was extended to identify a β-ketoacyl-acyl carrier protein synthase III-like quinolone synthase from *P. aeruginosa* that contributes to the biosynthesis of high levels of 4-hydroxycoumarin by *E. coli* [77].

### Peptides

Enzymatic hydrolysis of proteins generates mixtures of peptides. In contrast, purified carnosine (β-alanine-L-histidine) and antimicrobial peptides (e.g. gramicidin S, actinomycin, polymixin B, and vancomycin) are prepared from organisms that contain higher amounts compared with other organisms [78] (Table 5). Carnosine is synthesized from H-β-Ala-NH_2_ using β-aminopeptidase expressed by *E. coli* and *Pichia pastoris* [79]. Physiologically active polypeptides such as ε-poly-L-lysine (ε-PL) and poly-γ-glutamic acid (γ-PGA) are produced by microbial fermentation. ε-PL is characterized by the peptide bond between the α-carboxyl and ε-amino groups of 25–35 L-lysine residues [80] and is produced by secretory fermentation of *Streptomyces* strains isolated from soil [81]. The yield of ε-PL was enhanced using genome shuffling [82]. γ-PGA is an unusual anionic polypeptide comprising D/L-glutamate monomers polymerized through γ-glutamyl bonds [83]. γ-PGA is produced by *Bacillus* strains from glutamate or glucose [84,85]. *B. subtilis* was engineered to increase γ-PGA production by overexpression of γ-PGA synthetase [86] or by deletion of γ-PGA-degrading enzymes [87]. *B. amyloliquefaciens* was engineered to enhance γ-PGA production by heterologous expression of the *Vitreoscilla* gene (*vgd*) encoding hemoglobin to overcome the low concentration of dissolved oxygen [67,88]. In contrast, Chao et al. developed a γ-PGA-producing *E. coli* strain by heterologous expression of γ-PGA synthetase and glutamate racemase from *B. licheniformis* or *B. amyloliquefaciens* [89].

**Table 5:** **Enzymatic conversion and microbial fermentation of peptides**

**Compounds**	**Strains**	**Types** ^**a**^	**Genetic engineering** ^**b**^	**Substrates and components**	**Maximum yield**	**Scale**	**Reference**
Carnosine	*E. coli* or *Pichia pastoris*	E	**He:** *Ochrobactrium anthropi* β-aminopeptidase or *Sphingosinicella xenopeptidilytica* L-aminopeptidase-	H-β-Ala-NH_2_	4.5 g/L	200 mL	Heyland et al., 2010 [79]
			D-amidase				
ε-poly-lysine (εPL)	*Streptomyces* strains	F	**He:** Genome shuffling	Glucose	24.5 g/L	3 L	Li et al., 2013 [82]
poly-gamma-glutamate (γ-PGA)	*Bacillus subtilis*	F	**Oe:** γ-PGA synthetases	Xylose	9.0 g/L	50 mL	Ashiuchi et al., 2006 [86]
				Arabinose			
				Glutamate			
		F	**De:** γ-PGA degradation enzymes	Glutamate	48 g/L	20 mL	Scoffone et al., 2013 [87]
	*Bacillus amyrolique-haciens*	F	**He:** *Vitreoscilla* hemoglobin	Sucrose	3.5 g/L	100 mL	Zhang et al., 2013 [67]
		F	**He:** *Vitreoscilla* hemoglobin	Sucrose	5.1 g/L	100 mL	Feng et al., 2014 [88]
			**De:** cwlO and epsA-O cluster				
	*E. coli*	F	**He:** *Β. licheniformis* γ-PGA synthetases, glutamate racemase	Glutamate	0.65 g/L	100 mL	Cao et al., 2013 [89]
		F	**He:** *Β. amyloliquefaciens* γ-PGA synthetases, glutamate racemase	Glucose	0.52 g/L	100 mL	
Glutathione	*E. coli*	E	**De:** Single genes related to ATP regenerating activity	Glucose, Glutamate, Cysteine, Glycine	2.9 g/L	1 mL	Hara et al., 2009 [90]
	*S. cerevisiae*	E	**Oe:** GCS, GS	Glucose, Glutamate, Cysteine, Glycine	0.8 g/L	20 mL	Yoshida et al., 2011 [91]
	*S. cerevisiae*	F	**Oe:** GCS	Glucose	168 nmol/OD_600_	300 mL	Suzuki et al., 2011 [92]
			**De:** pep12				
	*S. cerevisiae*	F	**Oe:** GCS, Sulfate assimilation pathway genes	Glucose	43.9 mg/L	20 mL	Hara et al., 2012 [93]
Alanyl-glutamine	*E. coli*	E	**He:** *B. subtilis* L-amino acid α-ligase	Alanine, Glutamate	4.7 g/L	1 mL	Tabata et al., 2005 [94]
	*E. coli*	F	**He:** *B. subtilis* L-amino, α-ligase, L-alanine dehydrogenase	Glucose	24.7 g/L	2 L	Tabata et al., 2007 [95]
			**De:** dipeptidases, aminopeptidases				
	*E. coli*	E	**He:** *Sphingobacterium siyangensis* α-amino acid ester acyltransferase	L-alanine methyl ester hydrochlorid, Glutamine	79.3 g/L	300 mL	Hirao et al., 2013 [96]
Dipeptides	*E. coli*	E	**He:** *Ralstonia solanacearum* RSp1486a	Amino acids, ATP, MgSO_4_	2.9 g/L (Phe-Cys)	500 μL	Kino et al., 2008a [97]
		E	**He:** *B. licheniformis* BL00235	Amino acids, ATP, MgSO_4_	1.2 g/L (Met-Ala)	1.6 mL	Kino et al., 2008b [98]
		E	**He:** *B. subtilis* RizA	Amino acids, ATP, MgSO_4_	0.8 g/L (Arg-Ser)	300 μL	Kino et al., 2009 [99]

^a^Production types: E, Enzymatic production including permeable cell conversion; F, Fermentation.

^b^Types of genetic engineering: He, Heterologous expression; Oe, Overexpression of self-cloning gene(s); De, Deletion.

The tri-peptide glutathione, which is the most abundant antioxidant, thiol-containing compound among organisms [100], is produced enzymatically and by microbial fermentation. Glutathione is synthesized from glucose via glutamic acid, cysteine, and glycine through two consecutive ATP-consuming reactions catalyzed by ATP consuming two enzymes: γ-glutamylcysteine synthetase (GCS) and glutathione synthetase (GS). Enzymatic conversion of these substrates to glutathione was developed using permeabilized *S. cerevisiae* or *E. coli* overexpressing GCS and GS [91,101,102]. ATP regeneration is critical for improving the yields of glutathione, and a permeable cellular ATP-regenerating system was studied to provide an economical supply of ATP [101]. However, the efficiency of ATP regeneration for glutathione production is low [101]. To address this problem, the products of genes that increase ATP regeneration were systematically identified by generating *E. coli* mutants each with single deletions of *nlpD*, *miaA*, *hcp*, *tehB*, *nudB*, *glgB*, *yggS*, *pgi*, *fis*, *add*, *rfaB*, *ydhL*, or *ptsP* [90] from a single-gene deletion mutant library using high-throughput measurements of ATP regenerating activity [103]. Certain deletion mutants synthesized increased levels of glutathione [102]. These genes were classified into the following groups: (1) glycolytic pathway-related genes, (2) genes related to degradation of ATP or adenosine, (3) global regulatory genes, and (4) genes with unknown contributions to ATP regeneration. In contrast, improving ATP generation enhanced the enzymatic synthesis of glutathione by *S. cerevisiae* of about 1.7-fold [91]. Industrial glutathione production mainly uses yeast fermentation, because enzymatic synthesis of glutathione requires addition of substrates. Overexpression of GCS is critical for enhancing glutathione fermentation [92,93]. Enhancement of cysteine synthesis by engineering of sulfate metabolism also improved glutathione production [93]. In contrast, the overexpression of the transcription factors YAP1 and MET4 increased glutathione production [104-106]. Kiriyama et al. developed a fermentation system to efficiently produce extracellular glutathione by overexpression of a novel glutathione export ABC protein (Adp1p, Gxa1p) in *S. cerevisiae* [107].

A dipeptide L-alanyl-L-glutamine was enzymatically produced by *Sphingobacterium siyangensis* α-amino acid ester acyltransferase from L-alanine methyl ester hydrochlorid and glutamine [96]. Α *Bacillus subtilis* α-dipeptide synthase processing specificity for L-amino acids was discovered by Tabata et al. through *in silico* screening based on its amino acids similarity with members of the carboxylate-amine/thiol ligase superfamily, such as those that catalyze the synthesis of D-alanyl-D-alanine and γ-peptides [94]. They searched for the presence of an ATP grasp motif encoded by functional unknown genes in *B. subtilis*, because this motif is present in all enzymes of this superfamily [108,109] and showed that YwfEp exhibited dipeptide synthesis activity [94]. A variety of dipeptide synthases were subsequently identified using *in silico* screening based on amino acid sequence similarities to YwfEp [97-99]. Such screening approaches are useful for identifying peptide synthases with different substrate specificities. A microbial dipeptide fermentation system was developed by introducing the gene encoding YwfEp into *E. coli* and achieved [95].

### Polyphenols

Polyphenols such as phenolic acids, stilbenes, and flavonoids are secondary metabolites present in plants [110]. Polyphenols were traditionally extracted from plant sources using solvents or were chemically synthesized. Moreover, these methods are expensive and may be detrimental to the environment [111]. Recently, a metabolic engineering approach makes possible effective production of bio-based polyphenols. Phenolic acids are simple polyphenols. For example, ferulic acid and caffeic acid are produced by genetically engineered *E. coli* strains [65,112] (see “Aromatics” section). These phenolic acids, which form the skeletal structures of complex polyphenols, stilbenes, and flavonoids are biosynthesized through further genetic engineering (see [113] for an excellent review).

The biosynthetic pathway of the complex polyphenols requires coenzyme A (CoA)-esterified cinnamates and malonyl-CoA. Recently, the stilbene resveratrol was biosynthesized at high yields (2.3 g/L) by an *E. coli* strain [114] that was genetically engineered to enhance the production of malonyl-CoA to increase the supply of malonyl-CoA, which is used to synthesize fatty acids (see [115] for a review). Such metabolic engineering may further improve production. For example, the shikimic acid pathway, which produces phenylalanine and tyrosine as starting materials in the de novo production of polyphenols, would serve as a target. Actinomycetes may serve as useful hosts for producing aromatic amino acids. Further, *Aspergilli*, for which genetic engineering tools are available, may serve as promising hosts for producing antibacterial polyphenols, because *Aspergillus oryzae* was used to produce fine chemicals [116,117].

In general, producing high yields of polyphenols by microorganisms is difficult, because these compounds are strong antioxidants, and some are antibacterials, antifungals, or both [118]. Therefore, further improvements require using insensitive hosts and the development of an on-site recovery method for continuous fermentation. A membrane-purification process, which concentrates a target compound, would be implemented in such an on-site recovery and fermentation system. The combination of bio-based and engineering-based improvements would be required for producing high yields of polyphenols.

### Oligosaccharides

Oligosaccharides and rare sugars derived from the hydrolysis of plant polysaccharides are functionally diverse. Such oligosaccharides are categorized according to their monosaccharide subunits. For example, fructo-, xylo-, and gentio-oligosaccharides consist of short chains of fructose, xylose, and glucose, respectively, and are produced by enzymatic hydrolysis of extracts of natural sources, because they are difficult to synthesize *de novo* using microorganisms. For example, xylo-oligosaccharides are produced from xylan by enzymatic hydrolysis [119,120]; however, the quality and quantity of products strongly depend on the source compared with *de novo* synthesis.

Because of this, bio-based fermentation is under study. For example, the thermophilic fungus *Sporotrichum thermophile* and a genetically engineered *E. coli* strain produce fructo-oligosaccharides [121] and inulo-oligosaccharides (derived from insulin) [122], respectively. These bioconversions are advantageous, because the host cells do not metabolize the oligosaccharide products. Moreover, the specific functional monosaccharide L-arabinose was prepared from xylose [123]. Further, the microbial fermentation of 2′-fucosyllactose from lactose by a genetically engineered *E. coli* strain has been reported [124]. 2′-Fucosyllactose is a functional oligosaccharide present in human milk and protects newborns against infection by enteric pathogens [125].

The production of oligosaccharides requires the decomposition of polysaccharides or the polymerization of monosaccharides. Polymer-producing microorganisms would serve as promising hosts in a strategy based on the decomposition of polysaccharides. For example, the halophilic cyanobacterium *Arthrospira platensis* produces spirulan, which is an inhibitor of enveloped virus replication [126]. Moreover, it produces glycogen by fixing carbon dioxide, and the glycogen content reaches 65% of dry cell weight under optimum conditions [127]. Such photosynthetic microorganisms would serve as promising hosts for the de novo production of oligosaccharides. Specifically, the genetic engineering of microorganisms that produce polysaccharide-degrading enzymes, glycosyltransferases, or both may facilitate attaining this goal. In addition to fermentation, such polysaccharide-accumulating microorganism would be useful as a sugar source for bio-based production. In the future, combinations of polysaccharide-producing microorganism and decomposing, transferase-producing microorganism, or both would improve the bio-based production of oligosaccharides.

### Conclusion and future perspectives for the production of fine chemicals

Synthetic bioengineering employs molecular genetic approaches to engineer metabolic pathways to enhance the biosynthetic capabilities of well-characterized host strains to produce fine chemicals. These efforts include identifying the genes in plants and microbes encoding enzymes that catalyze the reactions of interest. Converting bio-based production of fine chemicals from enzymatic reactions to microbial fermentation reduces costs, because the latter uses less expensive substrates. Computational approaches are essential for synthetic bioengineering to increase yields, and an important aspect of designing strategies is to identify the initial key enzymatic reactions of a biosynthetic pathway (Figure 1). Using bioinformatics to mine genome and transcriptome data is the method of choice to identify novel enzymes and biosynthetic pathways to generate a wide range of compounds [128,129]. Sequence comparisons of putative and authentic genes allow the prediction of catalytic homologs and motifs with potentially new functions. Structural analyses such as active site modeling and docking simulation are alternative approaches. The availability of high-throughput sequencing technology and improved computational resources should accelerate synergy between bioinformatics and structural analyses to identify key enzymes from the vast reservoir of genetic and environmental data [130,131].

Once key enzymes are identified, one can move to pathway design and optimization for microbial production of the target compound. Several computational tools are available, such as BNICE [132], FMM [133], RetroPath [134], and DESHARKY [135] and M-path [136] for designing *de novo* metabolic pathways. These resources provide different views of metabolic pathways for microbial production that are generated using the enormous amount of information in metabolic pathway databases such as KEGG [137], MetaCyc [138] and BRENDA [139].

However, there are still limitations because of the computational complexity of possible combinations, and further improvements or other approaches will be required for precise and practical design of metabolic pathways. A standard method to optimize metabolic pathways is available as an alternative that is called flux balance analysis [140], which was developed to indicate how gene deletions and expression might be manipulated to distribute carbon toward chemicals of interest without inhibiting cell proliferation. Genome-scale models for some model organisms and an open-source platform (e.g. OptFlux) based on flux balance analysis allow the precise control of engineered metabolic pathways [141,142]. The extension of these tools will lead to further efficient production of fine chemicals by microbial cell factories.
